# Cartilage-Related Collagens in Osteoarthritis and Rheumatoid Arthritis: From Pathogenesis to Therapeutics

**DOI:** 10.3390/ijms24129841

**Published:** 2023-06-07

**Authors:** Ziwei Ouyang, Lei Dong, Feng Yao, Ke Wang, Yong Chen, Shufang Li, Renpeng Zhou, Yingjie Zhao, Wei Hu

**Affiliations:** 1 Department of Clinical Pharmacology, The Second Affiliated Hospital of Anhui Medical University, Heifei 230601, China; 2 The Key Laboratory of Major Autoimmune Diseases, Anhui Institute of Innovative Drugs, School of Pharmacy, Anhui Medical University, Heifei 230032, China

**Keywords:** collagen, cartilage, osteoarthritis, rheumatoid arthritis, cartilage repair

## Abstract

Collagens serve essential mechanical functions throughout the body, particularly in the connective tissues. In articular cartilage, collagens provide most of the biomechanical properties of the extracellular matrix essential for its function. Collagen plays a very important role in maintaining the mechanical properties of articular cartilage and the stability of the ECM. Noteworthily, many pathogenic factors in the course of osteoarthritis and rheumatoid arthritis, such as mechanical injury, inflammation, and senescence, are involved in the irreversible degradation of collagen, leading to the progressive destruction of cartilage. The degradation of collagen can generate new biochemical markers with the ability to monitor disease progression and facilitate drug development. In addition, collagen can also be used as a biomaterial with excellent properties such as low immunogenicity, biodegradability, biocompatibility, and hydrophilicity. This review not only provides a systematic description of collagen and analyzes the structural characteristics of articular cartilage and the mechanisms of cartilage damage in disease states but also provides a detailed characterization of the biomarkers of collagen production and the role of collagen in cartilage repair, providing ideas and techniques for clinical diagnosis and treatment.

## 1. Introduction

In mammals, collagen is the most abundant protein and one of the most abundant components of the extracellular matrix (ECM), accounting for more than one-third of the body’s protein tissue weight [1,2]. To date, approximately 28 types of collagen have been identified [3]. Collagen has a complex supramolecular structure, exists in highly diverse forms in different tissues, and has a range of biological functions [4]. In addition, collagen, as the basic structural component of connective tissue, plays a crucial role in maintaining its structural and biological integrity.

Osteoarthritis (OA) and rheumatoid arthritis (RA) are the most prevalent painful and disabling diseases worldwide [5]. OA is a chronic degenerative disease, and the main causes of OA are senescence, trauma, mechanical loading, and obesity [6]. RA is an inflammatory autoimmune disease in which the patient’s body’s immune tolerance balance is disrupted, producing large amounts of pro-inflammatory cytokines, matrix-degrading enzymes, and autoantibodies, ultimately leading to synovial inflammation and joint damage [7,8]. Destruction of articular cartilage represents a common pathological feature of OA and RA. In articular cartilage, numerous collagen subtypes have been identified; the major and most abundant collagens are type I, II, IX, and XI collagens, and the minor and less abundant collagens are type III, IV, V, VI, X, XII, XIV, XVI, XXII, and XXVII collagens [9,10]. These collagens have a significant role in maintaining the mechanical properties of articular cartilage and the stability of the ECM. During the pathophysiology of arthritis, the cartilage collagen network undergoes irreversible degradation, and the fragments formed by its degradation can be used as biomarkers of ECM degradation and disease progression [10,11]. It can overcome some limitations of current disease assessment methods and has been extensively studied [11]. Articular cartilage is a highly specialized connective tissue that lacks blood vessels, lymphatics, and nerves and is characterized by its limited capacity to heal after injury [12,13]. Currently, there is no cure for OA and RA. Existing treatments aim to reduce pain and symptoms, as well as improve joint functional capacity [14]. Therefore, the proper healing of articular cartilage injury has been one of the significant medical issues that needs to be addressed. In addition, collagen has the potential as a biomaterial for bone tissue engineering due to its abundance, biocompatibility, high porosity, ease of binding to other materials, ease of processing, hydrophilicity, low antigenicity, and restorability in vivo [2].

In this review, we searched the PubMed database using the terms ‘collagen’, ‘cartilage’, ‘RA’, ‘OA’, ‘biomarkers’, and ‘cartilage repair’. No timeframe was limited, and both human and animal studies were included. Ultimately, we present (1) a systematic description of collagens, (2) an analysis of the structural characteristics of articular cartilage and the mechanisms of cartilage damage in OA and RA, (3) the application of collagen metabolites as biomarkers of disease, and finally, (4) a detailed description of the role of collagen in cartilage repair.

## 2. Collagen

### 2.1. Collagen Structure and Biosynthesis Process

Collagen is defined by its unique dextro-triple helix structure, which consists of three left-handed polyproline-like helices, each with a (Gly-X-Y) repeated sequence, where X and Y are usually proline and hydroxyproline [15]. This repetitive sequence creates favorable hydrogen bonds that allow three polypeptides, called alpha-chains, to be assembled into a triple-helix collagen [16]. This unique structure is critical to the function of collagen in the body, providing elasticity, stability, and support to cells and tissues, strengthening bones, and forming basement membranes while influencing biological pathways, including cell signaling, motility, and differentiation [15,17].

The pathway of collagen biosynthesis is a complex multistep process from gene transcription to the secretion and aggregation of collagen monomers into functional protofibrils [4]. Most collagen genes display intricate exon–intron patterns, and in many cases, different mRNA species can be detected due to multiple transcription start sites, variable splicing of exons, or a combination of both [18]. Ribosome-bound mRNA is translated into procollagen molecules, which protrude into the rough endoplasmic reticulum for translation with the help of signal recognition domains recognized by the corresponding receptors [4,18]. Hydroxylation and glycosylation are the two major post-translational modifications (PTMs) of collagen that occur in the endoplasmic reticulum membrane, contributing to the triple helix’s thermal and mechanical stability and triple helix assembled forms of collagen, respectively [4,17]. Subsequently, procollagen is packaged into secretory vesicles within the Golgi and released into the extracellular space. During secretion into the extracellular space, procollagen is processed by proteolysis [4,18]. When entering the extracellular space, different molecular treatments are performed depending on the type of collagen in question and the supramolecular structure it must form in the tissue [4,9].

### 2.2. Collagen Turnover and Degradation

In normal tissue homeostasis, ECM synthesis and metabolism are in dynamic equilibrium, and collagen is slowly degraded and newly synthesized. The renewal rate of collagen varies significantly between different types. For example, the half-life of human cartilage collagen is approximately 117 years, that of spine-free disc collagen is 95 years, and that of skin collagen is 15 years [19,20]. Heinemeier et al. used a “14C bomb pulse” to measure collagen in the cartilage of the tibial plateau and found that the collagen matrix of human articular cartilage is essentially a permanent structure with no significant turnover even when disease occurs [21]. Therefore, collagen’s structural persistence needs to be considered when designing new cartilage repair strategies.

Collagen has a tightly wound triple helix and a complex supramolecular structure, and only two members of the protease family can degrade collagen, namely MMP and cathepsins [22]. Collagen degradation pathways can be divided into extracellular and intracellular pathways. Extracellular collagen degradation is mainly mediated by MMP and cathepsin K. The C-terminal hemopexin domain of MMP is critical for collagen degradation because it recognizes and binds substrates and unravels the collagen structure to access the cleavage site [23]. Collagenolytic activity has already been noticed for MMP1, MMP2, MMP7, MMP8, MMP9, MMP13, MMP14, and MMP19 [24,25]. In contrast, cathepsin K directly targets triple-helix collagen without unfolding the helix and may be the most effective protease for extracellular collagen degradation [26]. Intracellular collagen degradation is associated with lysosomes, which contain a series of cathepsins, including cathepsins B, D, K, and L, that cleave collagen into low molecular weight peptides [22].

### 2.3. Classification of Collagen Types

At least 46 different collagen genes have been identified in vertebrates, which can encode 28 different collagen proteins [27]. These collagens are denoted by Roman numerals (I–XXVIII) based on the chronological order of their discovery, using Greek letters to identify chains, bands, and higher molecular weight fractions [3,4,28]. Collagens can be homotrimers or heterotrimers, such as [a1(II)]_3_ for type II collagen consisting of three identical alpha chains, and a1(IX)a2(IX)a3(IX) for type IX collagen consisting of different genetically encoded alpha chains of the same collagen type [29,30]. In addition, a single collagen type may have a composition with multiple chains, such as a1(V), a2(V), and a3(V), for type V collagen [31]. Different types of collagens and their structures are essential to provide elasticity, stability, and strength to tissues and organs.

**Figure 1:**
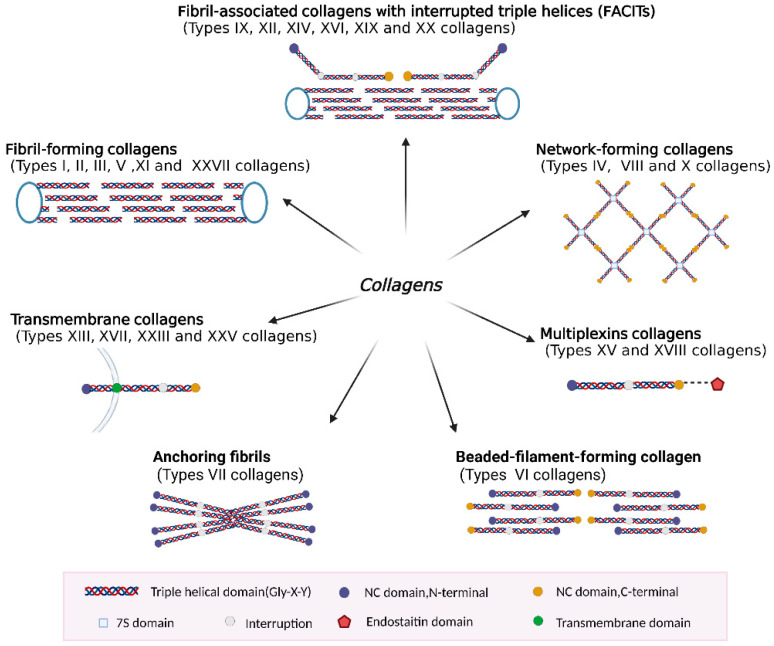
Classification of collagen types based on structural organization. Seven groups of collagens have been described: fibril-forming collagens, fibril-associated collagens with interrupted triple helices (FACITs), network-forming collagens, multiplexins collagens, transmembrane collagens, anchoring fibrils, and beaded-filament-forming collagen.

Based on their domain structure and macromolecular assembly, several groups of collagens have been described: fibril-forming collagens, fibril-associated collagens with interrupted triple helices (FACITs), network-forming collagens, multiplexins collagens, transmembrane collagens, anchoring fibrils, and beaded-filament-forming collagen (Figure 1).

The classical fibril-forming collagens are the most abundant in vertebrates and include type I, II, III, V, XI, and XXVII collagens [32]. They play a structural role by contributing to tissues’ molecular structure, shape, and mechanical properties. Fibrillar collagens are large molecules that consist of over 1000 residues per polypeptide chain in an uninterrupted (Gly-X-Y) repetitive sequence, forming a long triple helix that is approximately 300 nm long and ~1.5 nm in diameter [33]. Long and smooth protofibrils have alternating gaps and overlapping regions every 67 nm. This 67 nm structure is called the D-period, which is associated with the tensile strength of the bone, the stiffness of the extracellular matrix, and other biomechanical properties of the tissue [3,33].

The fibril-associated collagens with interrupted tripled helices (FACITs) include type IX, XII, XIV, XVI, XIX, and XX collagens [34]. The FACITs do not form fibers themselves but are associated with the surface of collagen fibers [3]. The FACITs regulate collagen fibril formation and size, as well as direct and control cellular organization in the extracellular matrix. The primary role of FACITs is to ensure the integrity and stability of the extracellular matrix and its fibrillar collagen network [34].

Network-forming collagens are non-fibrillar collagens that aggregate linearly or laterally to form open networks, mainly including type IV, type VIII, and type X collagens [35]. Network formation of collagen with disruption of the Gly-X-Y sequence is a common feature. This might help reduce the stiffness of collagen molecules, which provides more spatial freedom to the molecules and promotes superhelixes.

Type XV and XVIII collagens are classified as members of the multiplexin collagen superfamily, which are widely distributed basement membrane components [36,37]. These matrix molecules are commonly expressed in various vascular and epithelial basement membranes throughout the body [38]. Their structural features are mainly HS/CS side chains and a central collagen triple helix region with multiple interruptions flanked by an N-terminal laminin-G-like/platelet-1 sequence and a C-terminal endothelial repressor structural domain [36,37].

Collagens XIII, XVII, XXIII, and XXV are members of a subfamily called transmembrane collagens (MACITs) with an interrupted triple-helix structure. MACITs are type II transmembrane proteins that consist of a short cytosolic domain, a transmembrane domain, and a large extracellular ectodomain [39]. They are expressed in a variety of tissues and cells characterized prominently by cell adhesion [40].

Anchoring protofibrils are long centrosymmetric crossband protofibrils that are thought to ensure attachment of certain epithelial basement membranes to the underlying stromal matrix. Collagen VII is the main component of the anchoring protofibril, which consists of a very long triple-helix region flanked by the non-triple-helix of the amino-terminal NC-1 and carboxy-terminal NC-2 structural domains [41].

Collagen VI is a beaded filament-forming collagen commonly found in connective tissue, which includes joint cartilage, kidneys, tendons, cornea, and skin. It has an extensive filamentous network of collagen fibrils and is usually enriched in the pericellular region [42,43]. Collagen VI is a main component that plays a key role in defining the mechanical properties of the PCM of the surrounding chondrocytes [43].

### 2.4. Types of Collagens in Articular Cartilage

Numerous collagen subtypes have been identified in articular cartilage, such as type I, II, III, IV, V, VI, IX, X, XI, XII, XIV, XVI, XXII, and XXVII collagens (Table 1). They have different contents and distributions in the articular cartilage, but all play a major role.

Type I collagen is a heterotrimeric collagen consisting of two α1(I) chains and one α2(I) chain, and it is most abundant in vertebrates and expressed in almost all connective tissues [44]. It interacts with other collagen proteins of the ECM to form various tissue scaffolds, including bone, ligaments, tendons, skin, and blood vessels, giving them load-bearing mechanical properties [51]. Osteogenesis imperfecta (OI) is a genetic disorder most often caused by abnormalities in the synthesis or processing of type I collagen [52]. In articular cartilage, it is highly expressed mainly in fibrocartilage, also present in elastic cartilage. Type I collagen in hyaline articular cartilage indicates damage or a pathological state of the cartilage and can be found when ineffective fibrocartilage repair tissue fills the defect, ultimately leading to osteoarthritis [53].

Type II collagen accounts for 90–95% of hyaline cartilage collagen, interwoven with proteoglycan aggregates of hyaluronic acid, aggregated glycan core protein, and GAGs [16,54]. Type II collagen co-polymerizes with type IX, XI, XII, and XIV collagens to form a collagen network that provides cartilage tensile strength [55]. Type II collagen is enzymatically and mechanically degraded in the articular cartilage of OA and RA [56]. In particular, MMP-1 and MMP-13 cleave the type II collagen triple helix at the site between residues 776 and 778, and MMP-3 cleaves the denatured collagen within the telopeptide [45]. Type II collagen is also an important extracellular signaling molecule that regulates the proliferation, metabolism, and differentiation of chondrocytes [57].

Type III collagen is a homotrimer of three α1(III) chains. It accounts for approximately 5–20% of all collagens in the body [58]. Type III collagen is a major structural component of hollow organs, such as the great vessels, uterus, and intestine, providing tensile strength and integrity to organs [9,58]. Collagen III frequently co-assembles with collagen I to form heterotypic type I/III protofibrils and is thought to control protofibril diameter and participate in collagen cross-linking [59]. Some studies confirmed that type III collagen deposition occurs in adult articular cartilage, is more pronounced in OA joints, and is a potential marker of matrix repair or pathobiology [60]. Interestingly, most of the type III collagen molecules present in cartilage were found to be not fully processed and to contain disulfide-bonded N-terminal pre-peptides. This suggests that type III collagen can be easily extracted under natural conditions by the digestion of human articular cartilage by matrix degrading enzymes (MMP3) [46]. Type III collagen has been shown to be present in mesenchymal condensation, which precedes cartilage formation and osteogenesis and has a key role in bone development. Furthermore, its mutation can cause the dominantly inherited connective tissue disease vascular Ehlers-Danlos syndrome (EDS) [61].

Type IV collagen is a network-forming collagen that is a major component of the basement membrane (BM) of all organs [62]. It is a triple-helix structure composed of six different α-strands (α1, α2, α3, α4, α5, α6) in subtypes including α1α1α2(IV), α3α4α5(IV), and α5α5α6(IV) [63]. In articular cartilage, type IV collagen is normally present in the PCM of healthy cartilage tissue and is absent in degenerated and fibrotic cartilage tissue [63]. Articular cartilage is an avascular structure. Therefore, subtypes α1α1α2(IV) and α3α4α5(IV) of type IV collagen with unique anti-angiogenic properties may be involved in the temporal control of angiogenesis and cartilage homeostasis during cartilage repair [64]. It was found that MMP-2, and MMP-9 are the main enzymes involved in the degradation of type IV collagen [44].

Type V collagen is a heterotrimer consisting of one α1(V) chain and two α2(V) chains. It is essential for the formation of collagen fibrils. It usually forms heterofibrils with type I and III collagens, and can also be assembled into type V/XI collagen, an essential component of collagen protofibrils in vertebrates [47,65]. MMP-2- and MMP-9-mediated degradation of type V collagen in ankylosing spondylitis (AS) and its type V collagen fragment (C5M) can be used as biomarkers for AS [47]. One of the series of connective tissue diseases caused by mutations in type V collagen is the classic EDS [66]. Type V collagen is a key component in maintaining the structural integrity of the cartilage skeleton and plays a critical role in promoting the adhesion and proliferation of chondrocytes and osteoblasts. Studies have shown that type V collagen-stimulated adipose-derived stem cells (ADSCs) increase type II collagen synthesis and reduce apoptotic chondrocytes, suggesting that ADSC/Col V may be a therapeutic target for the treatment of osteoarthritis [67].

Type VI collagen accounts for 1% of the total collagen in adult articular cartilage and is mainly enriched in PCM and involved in chondrocyte attachment and integrity [68,69]. Type VI collagen interacts with various key ECM components, including fibrillar collagens type I and II, basement membrane type IV collagen, and fibronectin [70]. Thus, type VI collagen is likely to act as an interface between the interchondral matrix and chondrocytes and may be involved in cell anchoring and stromal cell signaling [69]. In articular cartilage, type IV collagen is present in the PCM of healthy cartilage tissue and is required to regulate pericellular matrix properties, chondrocyte swelling, and mechanical transduction in articular cartilage [71]. Collagen VI-deficient mice have reduced mechanical properties of the PCM, and this leads to the accelerated development of hip OA, which also leads to mitochondrial dysfunction and Ca^2+^ dysregulation, affecting muscle cell metabolism and mechanical transduction [71,72].

Type IX collagen is a heterotrimer consisting of α1(IX), α2(IX), and α3(IX) chains, which are mainly found in articular cartilage. Stefan Carlsen et al. found that a lack of type IX collagen in cartilage leads to the increased accessibility of structures to antibody binding, thus making joints more susceptible to inflammatory attack [73]. In mice, the knockdown of collagen IX resulted in severe disruption of the growth plate, abnormalities in the hypocellular zone, and chondrocyte shape [74]. These findings highlight that type IX collagen is a protein that is important for cartilage integrity and stability [73,74].

Type X collagen consists of three identical α1(X) chains, is synthesized by hypertrophic chondrocytes, and is present only in the calcified areas of hypertrophic cartilage and articular cartilage [75]. As the most widely used marker of chondrocyte hypertrophy, type X collagen is normally expressed in human OA cartilage, especially near lesions, but not in healthy human articular cartilage [75]. Type X collagen is also a reliable marker of new bone formation in articular cartilage. There is a spatial and temporal correlation between the synthesis of type X collagen and the onset of endochondral ossification, and it promotes endochondral ossification by regulating matrix mineralization and compartmentalizing matrix components [76].

Type XI consists of three different chains, α1(XI), α2(XI), and α3(XI), which are expressed mainly in cartilage [77]. Type XI collagen polymerizes to form the core of type II collagen fibers, serves as a template for type II collagen fiber production, and regulates the diameter of type II protofibrils in cartilage [77]. Type XI collagen has been identified as an ECM component with strong chondrogenic properties. In chondrocytes, collagen XI promotes cell proliferation and matrix production and inhibits matrix degradation [78]. Mutations in the gene encoding type XI collagen lead to changes in the matrix phenotype and cellular behavior that affect the biomechanical and functional properties of developing joints, leading to premature OA [79].

Type XII collagen, assembled from three identical α1[XII] chains, is an important regulator of tendon and ligament fiber production and fibrous organization, mainly in areas of cartilage with a more organized fibrous orientation. In addition, it plays a role in promoting the alignment or stabilization of this organization, thereby creating a matrix capable of withstanding gravity [80]. Regarding biological function, type XII collagen has been implicated in fibril formation, cell adhesion, fibrosis, and osteogenesis, and areas of high mechanical stress may act as protectors of tissue integrity, and mutations in collagen XII are the etiology of human connective tissue pathology and can cause congenital Ullrich disease or Bethlem myopathy [81]. It was found that, during osteoblast development, collagen VI/XII complexes are formed, which are essential for the formation of communication cell networks during bone formation [82].

Type XIV collagen is structurally similar to type XII collagen and is assembled from three identical α1[XIV] chains, usually present in areas of high mechanical stress. In dense connective tissue and bone, type XIV collagen regulates fibrillogenesis and maintains tissue integrity and mechanical properties [83]. 

Type XVI collagen has been identified in the territorial matrix of chondrocytes, and its primary function is to organize the ECM through stabilizing collagen fibers, anchoring micro-fibers, and mediating intracellular signals that affect cell adhesion, proliferation, invasiveness, and the formation of focal adhesions [10,84]. Type XVI collagen can interact with and activate the α1β1 integrin; therefore, type XVI collagen can also enter cells by triggering signals and inducing integrin-mediated cellular responses, such as spreading and changes in cell morphology [85].

Type XXII collagen consists of a homotrimer of three identical α1(XXII) chains. Type XXII collagen is expressed at the myotendinous junction and joint surface of articular cartilage and is associated with the extracellular matrix in cartilage [86]. Type XXII collagen is uniquely localized at tissue junctions in muscle, tendon, heart, articular cartilage, and skin, and it will be a marker for studying the formation of tissue junctions during development and regeneration, as well as identifying pathological processes [87].

Collagen XXVII consists of three identical α1(XXVIII) chains and is expressed primarily in developing cartilage tissue and, to a lesser extent, in other tissues [88]. Type XXVII collagen synthesis partially overlaps with type X collagen synthesis, precedes the transition from cartilage to bone, and is associated with cartilage calcification [89]. Expression of type XXVI collagen in chondrocytes is regulated by factors SOX9 and LC-Maf [88,90].

## 3. Role of Collagen in Cartilage Damage 

### 3.1. Collagen Is the Main Component of the Cartilage Matrix

Articular cartilage is primarily a tissue without blood arteries, nerves, or lymphatic vessels that absorbs and buffers stress [12]. Typically, it is composed of a sparse distribution of chondrocytes and a dense extracellular matrix. Chondrocytes are the only cell type in cartilage tissue and are responsible for the synthesis and maintenance of the basic structure of articular cartilage ECM [91]. Cartilage ECM is mainly composed of type Ⅱ collagen (Col Ⅱ), proteoglycan (PG), and water. The ECM plays an important role in the morphogenesis and cellular metabolism of new tissues, endowing them with mechanical and biochemical properties [18]. Type II collagen accounts for 90% to 95% of collagens in the ECM and is interwoven with proteoglycan aggregates to form fibrils and fibers. Type I, IV, V, VI, IX, and XI collagens, and other minor collagens, are also present and contribute to the formation and stabilization of the type II collagen fiber network [54]. Proteoglycans are also the major macromolecules in the ECM that occupy the interfibrillar space of cartilage ECM and provide permeability properties to cartilage [54]. Under normal physiological conditions, cartilage ECM synthesis and metabolism are in dynamic balance, which is important for maintaining the integrity and functionality of cartilage tissue [92].

**Figure 2:**
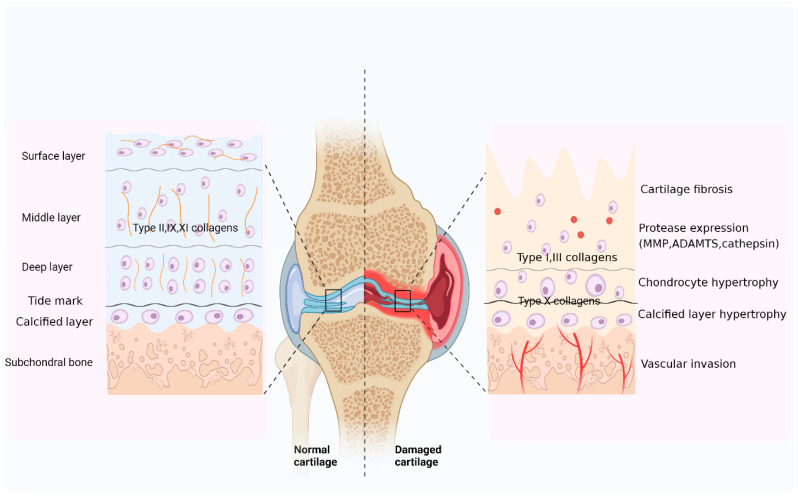
Microstructural and histopathological alterations in normal and damaged cartilage. Left: Normal articular cartilage is composed of chondrocytes and extracellular matrix. The chondrocytes are distributed in four horizontal layers (superficial, middle, and deep, with calcified layers) and show different morphologies. Right: In injured articular cartilage, the inflammatory cytokines, as well as the expression of proteases, are elevated, and the cartilage shows a fibrosis and hypertrophy phenotype, as well as articular cartilage fracture, calcified layer hypertrophy, and vascular invasion.

The articular cartilage zone can be divided into four levels of organization from the articular surface to the bone marrow cavity based on the arrangement of cells and matrix fibrils (Figure 2 left side, normal cartilage). These levels include the surface layer, the middle or transition layer, the deep layer, and the calcified cartilage layer [93,94,95]. The surface layer accounts for 10–20% of the total cartilage width and encloses elongated chondrocytes [54,96,97]. They realize the secretion of type II, IX, and XI collagen fibers, which are found parallel to the surface [96]. The following zone, termed the middle or transition layer, accounts for 40–60% of the cartilage volume and consists of rounded chondrocytes surrounded by collagen fibers with a more random, sloped organization [54,96,97]. The deep layer accounts for 30–40% of the cartilage volume, and chondrocytes are typically aligned in columns parallel to the collagen fibers. The thinnest layer is the calcified cartilage layer, which occupies 2–3% of the width. It contains a small number of hypertrophic chondrocytes and is separated from the rest of the area by calcified lines [54,96,97].

In addition, depending on the proximity to chondrocytes and the organization of collagen fibers, the cartilage matrix can be divided into different zones: the pericellular region, the territorial region, and the interterritorial region [54]. The pericellular matrix is a narrow tissue region surrounding the chondrocytes, and it contains mainly proteoglycans, as well as glycoproteins and other non-collagenous proteins [98]. The PCM serves as a transducer of both biomechanical and biochemical signals for the chondrocyte [94]. The territorial matrix surrounds the pericellular matrix, which is composed mainly of fine collagen fibers that form a basket-like network around the cells. This region is thicker than the pericellular matrix and may contribute to the resilience of the articular cartilage structure and its ability to withstand heavy loads. The interterritorial region is the largest of the three matrix regions, and it provides the greatest contribution to the biomechanical properties of the articular cartilage [9,54].

However, in disease states, the synthesis and metabolism of cartilage’s ECM is disrupted, and irreversible degradation occurs when degradation exceeds synthesis. With the development of injury, cartilage changes toward hypertrophy and fibrosis. Hypertrophic chondrocytes tend to express type X collagen, and fibrosis is manifested by the deposition of type I and III collagens [14]. In injured articular cartilage, the expression of proteases, including MMP, ADAMTs, and cathepsin, has been found to be elevated. In addition to this, the damaged cartilage also showed articular cartilage fractures, calcified layer hypertrophy, and vascular invasion [96] (Figure 2).

### 3.2. Collagen Is a Major Target of Cartilage Degeneration

**Figure 3:**
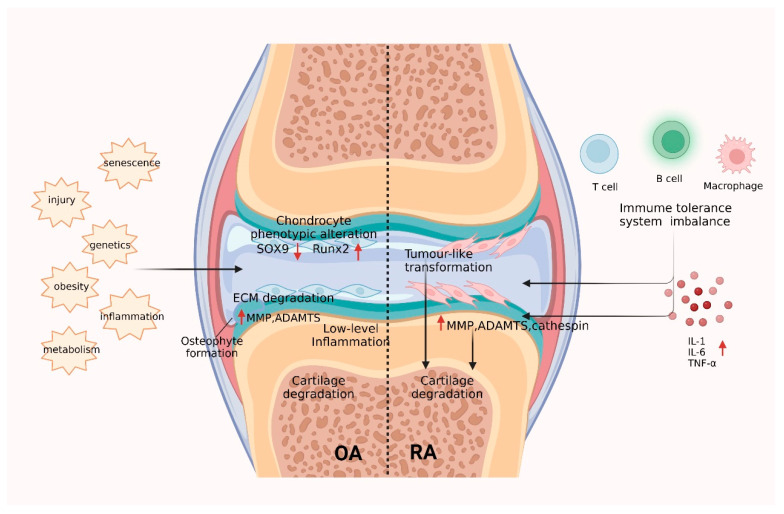
Mechanisms of cartilage damage in OA and RA. Left: In OA, different “stress” stimuli can activate chondrocytes, leading to loss of phenotypic stability and cartilage ECM degradation as well as inflammation. Right: In RA, the immune tolerance balance is disturbed, and a large number of inflammatory cytokines are produced, which leads to synovial FLS activation and ECM degradation. SOX9: Transcription factor SOX-9; Run2: Runt-related transcription factor 2; MMP: Matrix metalloproteinase; ADAMTS: A Disintegrin and Metalloproteinase with Thrombospondin motifs; OA: Osteoarthritis; RA: Rheumatoid arthritis.

Rheumatic and joint diseases, as exemplified by OA and RA, are among the most common and widespread painful and disabling pathologies across the globe [5]. Cartilage plays an active role in OA and RA by acting as a signaling scaffold harboring bioactive matrix components and soluble factors that interact with embedded chondrocytes and are released upon cartilage degradation. Cartilage damage is a crucial feature of RA and OA, and common mechanisms have been shown to play a role in OA and RA cartilage injury. The enzymes that ultimately mediate cartilage ECM degradation show substantial overlap between OA and RA, including many MMPs, metalloproteinases, and cathepsins [99,100]. Among the MMPs, MMP-1, MMP-2, MMP-3, MMP-9, MMP-13, and MMP-14 have attracted special interest [99,100]. Among them, the functional importance of MMP-13 in cartilage destruction has been demonstrated. MMP-13 is absent in normal adult cartilage and is specifically expressed in cartilage of OA and RA patients [101]. It is the major collagenase in cartilage and has the highest activity on type II collagen. In addition to MMP-13, metalloproteinases (A disintegrins and metalloproteinases with thrombospondin motif protein family, ADAMTS), ADAMTS-4, and ADAMTS-5 are most closely associated with cartilage damage, are involved in articular cartilage degradation, and are potential therapeutic targets for arthritis treatment [102,103]. In addition, it is essential to study the events that trigger cartilage damage and the differences between physiological cartilage remodeling and unbalanced cartilage destruction in OA and RA (Figure 3).

#### 3.2.1. Mechanisms of Collagen in OA Cartilage Injury

OA is an age-related chronic degenerative disease that results mainly from senescence, trauma, and mechanical stress but is also influenced by obesity, inflammation, genetics, and metabolism [6]. The main features of OA are progressive degeneration of the cartilage matrix, bone redundancy formation, subchondral osteosclerosis, and synovial inflammation [96]. In OA, different stress stimuli activate chondrocytes, causing a loss of phenotypic stability, cartilage ECM degradation, and low-level inflammation.

Senescence has been recognized as the most prominent risk factor for OA. With aging, many molecular pathways become unregulated, disrupting the dynamic balance of the ECM in cartilage and destroying cartilage structures. Senescence is characterized by permanent cell cycle arrest and the release of harmful pro-inflammatory molecules into the surrounding microenvironment, a feature known as the senescence-associated secretory phenotype (SASP) [104]. Another feature of senescence is mitochondrial dysfunction, which can increase cellular ROS levels to cause oxidative stress [105]. Oxidative stress can cause an imbalance of catabolic and anabolic signals in OA cartilage. Many SASP and some elevated ROS levels can be detected in articular cartilage and synovial tissue in OA, which can lead to the loss of articular cartilage type II collagen and cartilage destruction. In addition, the structure of collagen fibers changes with aging, affecting the collagen network’s stability and integrity [106].

Joint injury is a significant risk factor for osteoarthritis. In younger patients and highly active individuals, articular cartilage may be damaged by abnormal mechanical loading or trauma [107]. Joint injury may result in the release of inflammatory cytokines that induce chondrocytes to produce MMPs, aggregates, and other enzymes, leading to increased cartilage matrix degradation [108]. MMP-13 is the primary type II collagen-degrading collagenase involved in the degeneration of articular cartilage structures.

Obesity is another significant risk factor for knee OA. Obesity not only leads to increased joint loading but also enhances adipokine production in adipose tissue, which can lead to inflammatory or autoimmune diseases. Adipokines can also act as SASPs involved in the cellular senescence of chondrocytes [105,109]. Adipokines mainly include adiponectin, leptin, chemokines, and resistin, as well as tumor necrosis factor (TNF-α) and inflammatory cytokines (IL-6). Lipocalin is considered to be one of the adipokines associated with the pathogenesis of OA and is a potential catabolic mediator in OA. It increases NO and MMP-1, MMP-3, and MMP-13 levels in chondrocytes mainly through the AMPKJNK pathway in vitro, as well as causes an increase in type II collagen neoepitopes cleaved by collagenase in OA. This indicates the involvement of lipocalin in OA cartilage matrix degradation [110]. Moreover, lipocalin stimulates the increased expression of aggrecan, Runx2, and type X collagen, contributing to the change in chondrocytes towards the hypertrophic phase [111]. Leptin is the most widely studied adipokine, and it has been found to be highly expressed in human OA. Leptin has been shown to upregulate MMP-1 and MMP-3 production in OA cartilage and positively correlate with MMP-1 and MMP-3 in the synovial fluid of OA patients [112]. Treatment of chondrocytes with leptin promotes proliferation, differentiation, type X collagen production, and cytoskeletal remodeling via the RhoA/RhoA kinase (ROCK) pathway [113].

There is growing evidence that inflammatory pathways are associated with OA, but whether inflammation is a key trigger or a secondary phenomenon remains controversial. Inflammation is a chronic, aseptic, low-grade inflammatory state that triggers a cascade response of chondrocytes to a phenotypic shift toward hypertrophy, cartilage destruction, and bone remodeling by affecting chondrocyte metabolism. It also involves metabolic pathways as well as activation of the innate immune system [100,114].

#### 3.2.2. Mechanisms of Collagen in RA Cartilage Injury

RA is considered to be a systemic polyarticular chronic inflammatory autoimmune joint disease caused by complex interactions between genetic and environmental factors. This results in a disruption of the body’s immune tolerance balance and the production of large amounts of pro-inflammatory cytokines, matrix-degrading enzymes, and autoantibodies, ultimately leading to synovial inflammation and joint damage [8]. Chronic inflammation and cellular activation characterize the pathophysiology of RA, and fibroblast-like synoviocytes (FLS) are the key mediators of progressive stromal destruction [115]. In RA, synovial cells are activated to transform into a disease-specific, tumor-like, permanently imprinted phenotype [8,116]. Tumor-like transformation is a critical distinction between RA and OA, an essential feature of rheumatoid cartilage injury. The synovial immunopathology of RA has been well documented in that during the course of the disease, inflamed synovial cells, particularly FLS, attack the cartilage and cause its progressive destruction [115,116].

Articular cartilage destruction in RA is closely associated with the inflammatory environment. The synovial lining layer is the primary site of inflammation in RA. The resident cells here are FLS and synovial tissue macrophages (STM), which translate into overproduction of enzymes that degrade cartilage and bone, as well as cytokines that promote immune cell infiltration [117]. In patients with RA, the balance between pro-inflammatory and anti-inflammatory cytokines is disrupted and inflammatory factors, such as tumor necrosis factor (TNF)-α, interleukin (IL)-1β, and IL-6, are released in large amounts [118]. The inflammatory environment causes chondrocytes to secrete large amounts of matrix-degrading enzymes. The ECM synthesis metabolism balance is disturbed, leading to the degradation of the cartilage ECM from within, which is the central link leading to the destruction of cartilage structure [119].

Autoantibodies are an extensively studied topic in RA, and many autoantibodies have been identified as markers of RA. A variety of well-characterized autoantigens are present in patients with RA, such as guanosine proteins and peptides, components of articular cartilage (type II collagen), circulating serum proteins, enzymes, and other target proteins [120]. Autoantibodies against these autoantigens, such as rheumatoid factor (RF) and anti-citrullinated protein antibodies (ACPA), and increased expression of anti-collagen type II antibodies are present. In different mouse models, autoantibodies have been shown to induce arthritis. Collagen antibody-induced arthritis (CAIA) is induced by the injection of a mixture of anti-collagen type II antibodies, leading to inflammation and bone and cartilage erosion of the joint [121]. RF and ACPA are the two most representative autoantibodies in the diagnosis of RA, which help in the treatment and prognosis of RA [122].

## 4. Collagen Metabolites as Biochemical Markers for OA and RA

Collagen is the main structural component of the extracellular matrix and plays a key role in tissue development and regeneration. Collagen remodeling involves collagen synthesis (promoted by growth factors) and degradation (by proteases) [123]. Collagen remodeling is an integral part of normal tissue renewal, but excessive remodeling activity or its occurrence in abnormal locations is usually involved in tumors, arthritis, and many other pathological conditions [123]. The irreversible degradation of the cartilage collagen network is considered to be a key event in the pathophysiology of arthritis. Collagen is degraded by catabolic enzymes in response to inflammatory mediators, producing “fragments” that are released into the synovial fluid and systemic circulation [124]. Specific products of collagen formation and degradation can be used as biomarkers of ECM degradation and disease progression, and these biomarkers have the unique properties of dynamic changes, high sensitivity, and ease of measurement that can overcome some of the limitations of current disease assessment methods.

Conventional biomarkers of joint tissue turnover are biochemical indicators developed using common biochemical techniques, and they have been extensively studied [125]. The ECM of cartilage is mainly composed of type II collagen, and type I and III collagens are the main proteins of the periarticular soft tissues [11]. In OA and RA, the upregulation of pro-inflammatory cytokines and cartilage-degrading proteases, such as MMPs, as well as cell activation and differentiation, lead to the production of protein degraders [11,126]. Type II collagen is the most abundant protein in cartilage and is the most studied cartilage protein in terms of developing biomarkers. Type II collagen-derived fragments have been extensively investigated as potential markers of cartilage remodeling in OA and RA [127]. Different strategies have been developed, including the measurement of type II collagen pre-peptides or degradation products in biological fluids and tissue explants. Type II collagen is synthesized from procollagen consisting of C- and N-terminal pro-peptides (PIICP and PIINP), which have been widely recognized as biomarkers reflecting cartilage formation [125,128]. Urinary C-terminal telopeptide (uCTX-II), an MMP-derived type II collagen fragment, is currently the most promising biological OA marker that has been evaluated in various clinical studies [125,129]. Analytical studies found that uCTX-II assays were able to distinguish patients with OA from healthy controls, and the expression level of uCTX-II increased with disease progression [130,131]. C1M, a product of type I collagen MMP cleavage and a biomarker of soft tissue destruction, has proven its value in RA and OA [126,130]. MMP-mediated type III collagen (C3M) metabolites reflect soft tissue degradation and are implicated in the pain mechanisms of RA and OA [132]. Bay-Jensen et al. developed a new ELISA-specific ADAMTS-generated type III collagen fragment and identified the type III collagen neoepitope COL3/ADAMTS as a marker for early osteoarthritis [133].

Despite representing only a small fraction of the mature matrix, minor collagens still play important structural roles and have specific biological functions. In joint diseases, the progressive destruction of cartilage and the degradation of matrix components occurs, including these secondary collagens [10]. Thus, fragmentation molecules released from minor collagen are equally important biomarkers for detecting disease progression. It has been shown that many fine collagens of articular cartilage, such as IV, VI, IX, and X collagens, are susceptible to degradation by MMPs. MMP degrades type IV collagen, leading to the release of protein fragments into the circulation, quantified as biochemical markers of basement membrane turnover [134]. C4M is a serological marker of type IV collagen metabolism and has been shown to be a plausible biological marker of destructive synovitis growth in RA [135]. It is well known that type X collagen is a marker of phenotypic changes in chondrocytes toward hypertrophy. Y He et al. identified a new epitope of type IV collagen in urine samples from OA patients and demonstrated that Col10neo associated with hypertrophic chondrocytes could be used as a biochemical marker for the diagnosis of knee OA [75].

## 5. Collagen as a Tool for Cartilage Repair

Articular cartilage is a unique load-bearing connective tissue with a low intrinsic capacity for repair and regeneration. Due to its avascular nature and inability to overcome the inflammatory response, cartilage is susceptible to attack by pro-inflammatory factors and oxidative stress [12,13]. Articular cartilage has a very poor intrinsic healing capacity, and its repair is a significant clinical challenge. To date, no successful targeted therapy has been able to halt or even reverse the progression of cartilage damage. Current medications include NSAIDs for reducing inflammation and pain, anti-inflammatory–proton pump inhibitor drug combinations for patients on long-term NSAID therapy, and hormonal therapies to replace estrogen deficiency, which can only be treated palliatively [136,137]. Rehabilitation therapy (RT) helps to both improve patients’ level of function and independence and improve their quality of life, but only as a conservative treatment to reduce pain and not as a cure [138]. Therefore, cartilage repair strategies are necessary for many joint diseases. Several different methods have been used clinically to replace cartilage at the site of injury, including osteochondral autologous transplantation (OAT), osteochondral allograft transplantation (OCA), particulated articular cartilage implantation (PACI), microfractures (MF), and autologous chondrocyte implantation (ACI). However, none of these cartilage repair strategies has produced durable hyaline cartilage that meets functional requirements [139]. Therefore, further in-depth research is needed to optimize existing technologies or to develop entirely new therapeutic approaches.

Following the development of tissue engineering and the promising strategies for its repair, there is growing interest in the development of biocompatible and biodegradable materials for tissue regeneration. In addition to resorbable and non-resorbable synthetic materials, biomaterials with cartilage conduction properties based on natural polymers, especially collagen, have been developed [140]. Collagen is the most important tissue protein that plays a crucial role in the formation of the basic components of connective tissue, and its metabolism is connected to many physiological processes of biological adaptation and tissue regeneration. Collagen is a biologically derived protein that has been approved as an efficient bone tissue engineering material because it effectively retains bioactive molecular activity and contributes to the osteogenic differentiation of bone marrow mesenchymal stem cells (BMSCs) [141]. Collagen as a biomaterial has received much attention in biological applications for its excellent properties, such as low immunogenicity, biodegradability, biocompatibility, hydrophilicity, and ease of processing [142].

### 5.1. Collagen Derivatives for Cartilage Repair

Clinical drug therapy is often accompanied by considerable side effects, and in the absence of effective drug therapy, many patients turn to nutritional supplements and nutraceuticals, including collagen derivatives. Collagen derivatives are classified into three main categories based on the different degrees of hydrolysis of collagen: undenatured collagen (UC), gelatin, and collagen hydrolysate (CH) [143]. Evidence suggests that orally tolerated (OT) ingestion of collagen may impede the immune response against endogenous collagen and reduce inflammation [144]. Undenatured collagen type II (UC-II) is a nutritional supplement derived from chicken breast bone cartilage [145]. Studies have shown that UC-II treatment of patients with OA increases joint mobility and function and reduces pain [145,146]. In animal models, UC-II influences the humoral and cellular immune response through the secretion of anti-inflammatory factors by T regulatory cells, thereby inhibiting the immune response to type II collagen present in the extracellular matrix of articular cartilage [147,148]. All published clinical trials have found that oral supplementation with collagen derivatives or administration via intra-articular injection has the potential to reduce cartilage destruction or promote cartilage repair, but most clinical trials have been relatively short-term and small in size, so more independent and high-quality studies are needed to demonstrate their effects [149].

### 5.2. Collagen Matrices as Vehicles for Direct Drug Delivery

Drug delivery systems (DDSs) play an important role in avoiding growth factor and protein drug degradation. Collagen-based DDSs are available in various forms, including hydrogels, scaffolds, particles, microcapsules, or microspheres [142].

Hydrogels have structural and functional properties similar to most tissues and are among the most attractive candidates for the ECM. Collagen is the main component of the ECM, and collagen hydrogels provide an advantage to cells through their water-rich microenvironment and the continuous delivery of growth factor activity [150]. Type II collagen is a major component of articular cartilage and a promising material for repairing cartilage defects, playing a key role in chondrocyte function. Type I collagen, which is abundantly available and has been included in FDA-approved tissue-engineered structures, remains the most commonly used type of collagen in tissue-engineered scaffolds [151]. Pallabi Pal et al. studied the development of bone regeneration hydrogels based on collagen and elastin-like polypeptide (ELP) loaded with recombinant human bone morphogenetic protein-2 (rhBMP-2) and doxycycline, with mechanical properties suitable for osteogenesis [152]. The results showed that the drug-laden hydrogels possessed the greatest expression of the osteogenic markers, alkaline phosphatase and osteocalcin, compared to the drug-free hydrogels.

Collagen scaffolds are a common form of collagen material used for the slow release of drugs. Dongtak Lee et al. developed a biodegradable sequential drug delivery scaffold for bone regeneration [153]. A collagen-hydroxyapatite scaffold (CHAS) was used to dope PLGA microspheres encapsulated with ALN, and then BMP-2 was loaded onto the scaffold. The results suggest that the sequential delivery of BMP-2 and ALN from the scaffold has a synergistic effect on bone regeneration. Hilal Ahmad Rather et al. used a dual drug delivery scaffold of polycaprolactone–collagen fibers to deliver simvastatin and dexamethasone, which significantly promoted early osteogenesis and angiogenesis [154]. Moreover, the addition of other materials to the collagen-based scaffold can lead to specific functionalities. For example, heat-sensitive liposomes covalently attached to a collagen–hydroxyapatite scaffold can respond to thermal triggering to cause drug release [155].

Nano-engineered capsules or particles are one of the promising technologies for targeted drug release. The release characteristics can be controlled not only by container degradation and drug diffusion but also by pH changes, degradation enzymes, light, magnetic fields, or ultrasound [142]. The responsiveness of nanomaterials helps to reduce the dose and can improve the efficacy of drugs by delivering active substances with low bioavailability directly to the joints. Moreover, the small size and high surface area of nanomaterials can increase the solubility and intracellular uptake of active substances, properties that help address the limitations of conventional therapies [156]. Typically, collagen-based nano-engineered capsules are used as smart drug containers for the treatment of joint injuries, such as type I collagen microcapsules delivering gold nanoparticles (GNPs) [142]. It is also possible to guide the drug to precisely reach the articular cartilage by modifying type II collagen-targeting peptides on the surface of nanomaterials [157].

### 5.3. Collagen Matrices as Vehicles for Cell Delivery

The cell-based cartilage repair matrix is designed to provide anchoring support for the cells and to position them to the defect site at the time of implantation to prevent graft delamination and failure [158]. Collagen demonstrates excellent biocompatibility, negligible immunogenicity, specific interactions with growth factors and cell adhesion molecules, and biodegradability, as well as allows for inward cell growth and remodeling [142]. It has been used as an ideal scaffold for cartilage tissue engineering. ACI has been successful in cartilage repair, but it also has the disadvantages of insufficient supply of chondrocytes, differentiation of monolayer cultured chondrocytes, and periosteal hypertrophy. In recent years, stem cells, especially mesenchymal stem cells (MSCs), have emerged as an alternative cell type to circumvent some of the drawbacks of ACI [159]. MSCs can be isolated from autologous sources, such as bone marrow and adipose tissue, and are characterized by multiple differentiation potentials, a self-renewal ability, ease of acquisition, and low immunogenicity. They can differentiate into multiple lineages, including osteoblasts, adipocytes, and chondrocytes [160]. They also act as cellular regulators, endorsing tissue repair by secreting bioactive molecules [161]. MSC-based therapies have demonstrated the effectiveness of cartilage repair in animal and clinical studies. MSC was isolated from bone marrow and encapsulated in collagen microspheres to form MSC–collagen microspheres. These microspheres can serve as excellent cell delivery devices because of their injectability and ability to provide a protective growth and migration support matrix for MSCs. Moreover, MSCs can retain their stem cell properties when microencapsulated, survive and proliferate within the microspheres, migrate through the microspheres, and can be localized with good integration at the implantation site [158,162].

## 6. Conclusions

The many types of collagens present in articular cartilage are the main structural components of the ECM, providing support for cell growth and being responsible for the mechanical elasticity of the connective tissue. The collagenous cartilage matrix is the target of destructive processes in arthritic and degenerative joint diseases. In OA and RA, collagen degrades, producing “fragments” that are released into the synovial fluid and systemic circulation. Such fragments are biomarkers of ECM degradation and disease progression. Biochemical markers have received increasing attention for their ability to detect the early stages of the disease process, monitor the progression of destruction and predict the development of arthritis, and accurately and relatively quickly assess the efficacy of treatment. Therefore, the development of simple and reliable biomarkers of collagen metabolism is an essential goal for clinical research, and their development may provide new translational diagnostic tools for studying the effects of known drug targets on cartilage in preclinical or clinical settings. In addition, collagen also has excellent bioactivity, so it is not only a major target for matrix destruction and regeneration but also a major tool for cartilage repair based on collagen material delivery cells, growth factors, and drugs, which have received a lot of attention in recent years. Considerable potential exists for using collagen to repair cartilage damage in future studies.

## Figures and Tables

**Table 1:** Types of collagens that may be present in articular cartilage.

Collagen Type	Molecular Composition	Classification	Distribution in Articular Cartilage	Susceptible to Proteinases
Type I	[α1(I)]2α2(I)	Fibril-forming collagens	Fibrocartilage, Elastic cartilage	MMP-2 [44]
Type II	[α1(II)]3	Fibril-forming collagens	ECM of all zones	MMP-1,3, 13 [45]
Type III	[α1(III)]3	Fibril-forming collagens	PCM	MMP-3 [46]
Type IV	[α1(IV)]2α2(IV) α3(IV)α4(IV)α5(IV) [α5(IV)]2α6(IV)	Network-forming collagens	PCM	MMP-2, 9 [44]
Type V	α1(V)2α2(V)	Fibril-forming collagens	PCM	MMP-2, 9 [47]
Type VI	α1(VI)α2(V)α3(V) α1(VI)α2(V)α4(V) α1(VI)α2(V)α5(V) α1(VI)α2(V)α6(V)	Beaded Filament-Forming Collagen	PCM	MMP-2, 9 [3]
Type IX	α1(IX)α2(IX)α3(IX)	FACIT	Growth-plate cartilage	MMP-3, 13 [48]
Type X	[α1(X)]3	Network-forming collagens	Calcified zone and hypertrophic cartilage	MMP-1, 3, 13 [49]
Type XI	α1(XI)α2(XI)α3(XI)	Fibril-forming collagens	Articular cartilage	MMP-2 [50]
Type XII	α1[XII]3	FACIT	Cartilage with more organized fibril orientation	NA
Type XIV	α1[XIV]3	FACIT	Uniformly throughout the articular cartilage	MMP-13
Type XVI	[α1(XVI)]3	FACIT	Territorial matrix of chondrocytes	NA
Type XXII	[α1(XXII)]3	FACIT	Articular surface of joint cartilage	NA
Type XXVII	[α1(XXVIII)]3	Fibril-forming collagens	Proliferative Zone chondrocytes	NA

FACIT: fibril-associated collagens with interrupted triple helices; MMP: Matrix metalloproteinase; ECM: extracellular matrix; PCM: pericellular matrix; NA: Not available.

## Data Availability

Not applicable.
